# Modulation of CXCR3 ligand secretion by prostaglandin E_2 _and cyclooxygenase inhibitors in human breast cancer

**DOI:** 10.1186/bcr3115

**Published:** 2012-02-14

**Authors:** Holger Bronger, Sara Kraeft, Ulrike Schwarz-Boeger, Claudia Cerny, Alexandra Stöckel, Stefanie Avril, Marion Kiechle, Manfred Schmitt

**Affiliations:** 1 Department of Gynecology and Obstetrics, Technische Universität München, Ismaninger Str. 22, 81675 Munich, Germany; 2 Department of Pathology, Technische Universität München, Trogerstr. 16, 81675 Munich, Germany

## Abstract

**Introduction:**

In murine breast cancer models, the two interferon-gamma (IFN-γ) inducible chemokines and CXC-chemokine receptor 3 (CXCR3) receptor ligands, monokine induced by γ-interferon (CXCL9) and interferon-γ-inducible protein-10 (CXCL10) impair tumor growth and metastasis formation through recruitment of natural killer (NK) cells and tumor-suppressive T lymphocytes. In human breast cancer, CXCL9 mRNA overexpression correlates with the number of tumor infiltrating lymphocytes and predicts response to different chemotherapeutic regimens. Raising the intratumoral CXCR3 ligand concentration is therefore a possible way to enhance immune intervention in breast cancer. Little is known, however, about expression levels and regulation of these chemokines in human breast cancer. Since the inhibition of cyclooxygenases (COX) has been shown to reduce tumor growth and incidence of metastases in a lymphocytic and IFN-γ dependent manner, we argued that COX isoenzymes are a pharmacologic target to increase intratumoral CXCR3 ligand concentration in human breast cancer.

**Methods:**

CXCL9 was visualized in breast cancer specimens by immunohistochemistry, expression levels of CXCL9 and cyclooxygenases were determined by ELISA and western blotting, respectively. For regulation studies, Michigan Cancer Foundation-7 (MCF-7) and M.D. Anderson - Metastatic Breast 231 (MDA-MB 231) breast cancer cells were stimulated with IFN-γ with or without prostaglandin E_2 _(PGE_2_) or COX inhibitors (indomethacin, acetylsalicylic acid (ASA), celecoxib). CXCR3 ligand release from cells was measured by ELISA.

**Results:**

Within the tumor microenvironment, cancer cells are the major source of CXCL9. PGE_2 _impairs IFN-γ mediated CXCL9 and CXCL10 release from MCF-7 and MDA-MB 231 cells, and inhibition of endogenous cyclooxygenases by indomethacin or ASA correspondingly increases this secretion. Otherwise, high concentrations of the Cyclooxygenase-2 (COX-2) specific antagonist celecoxib have opposite effects and impair CXCL9 and CXCL10 release. In human breast cancer tissue specimens there is an inverse correlation between COX-2 overexpression and CXCL9 concentration, suggesting that the observed *in vitro *effects are of importance *in vivo *as well.

**Conclusions:**

Suppressing endogenous PGE_2 _synthesis by cyclooxygenase inhibition increases CXCL9 and CXCL10 release from breast cancer cells and is therefore a pharmacologic candidate to enhance intratumoral immune infiltration. Yet, to this end the unselective COX inhibitors ASA and indomethacin seem preferable to celecoxib that at higher concentrations reduces CXCR3 ligand release most probably due to COX independent mechanisms.

## Introduction

Successful immunotherapeutic strategies in cancer treatment require an effective infiltration of the tumor by tumor-suppressive immune cells. Immunotherapeutic approaches can therefore be impeded by an unfavorable composition of the intratumoral immune milieu: while regulatory T cells (T_regs_) and myeloid-derived suppressor cells (MDSCs) repress a successful immune intervention and promote tumor progression, natural killer (NK) cells and T helper (Th) 1 CD4^+^/CD8^+ ^lymphocytes are potent mediators of anti-tumor activity [1-3]. However, insufficient migration of this type of immune cells towards the tumor microenvironment will not allow attacking of cancer cells by these cells in patients with advanced tumors [4,5].

The CXCR3 chemokine receptor is preferentially expressed on the surface of NK cells or Th1 tumor-suppressive T lymphocytes and is responsible for their chemotactic recruitment into the tumor tissue [6,7]. Correspondingly, high intratumoral concentrations of the interferon (IFN)-γ-inducible chemokines CXCL9 and CXCL10, two of the CXCR3 ligands, are associated with increased immune infiltration and improved survival in patients with solid malignancies [8-13]. In human breast cancer, we and others have shown that a high expression of the *CXCL9 *mRNA correlates with an increased number of infiltrating lymphocytes and a better response to chemotherapy [14,15]. Furthermore, in a mouse model transfection of murine breast cancer cells with CXCL9 increases chemotactic T cell recruitment, impairs tumor growth, prevents lung metastasis formation, and prolongs survival [16]. Raising the intratumoral concentration of CXCR3 ligands is therefore a feasible therapeutic option to improve immune intervention. Still, origin and regulation of CXCR3 chemokines in human breast cancer are poorly understood.

A conceivable way to shift the tumor microenvironment to a more tumor-suppressive Th1 milieu is modulation of the cyclooxygenase (COX) system. Two isoenzymes, constitutively expressed COX-1 and inducible COX-2, are found in human breast tumors. COX-2 overexpression is associated with reduced infiltration of tumor-suppressive immune cells, and COX inhibition in turn enhances immunosurveillance [17-19]. Moreover, prostaglandin E_2 _(PGE_2_), the major product of COX in tumors, promotes tumor growth at least in part by reducing the activity of NK cells and expanding MDSCs and T_regs _[20,21]. In breast cancer models, both COX inhibition and PGE_2 _receptor antagonism suppress local tumor growth and metastatic spread in an IFN-γ and T cell- or NK cell-dependent manner [22-24].

In light of these findings, we explored whether components of the COX pathway would be pharmacologic candidates to enhance CXCR3 ligand concentration in human breast cancer. We now demonstrate that PGE_2 _inhibits IFN-γ induced CXCL9 and CXCL10 secretion from breast cancer cells and that, conversely, the COX inhibitors acetylsalicylic acid (ASA) and indomethacin augment this release. Inverse correlation of COX expression and intratumoral CXCL9 concentration in human breast cancer samples indicate the relevance *in vivo*. The COX-2-specific inhibitor celecoxib, however, has the opposite effects at higher concentrations implicating that the choice of the appropriate COX inhibitor for clinical use seems to be decisive. In summary, our results provide a mechanistic link between the COX pathway and a reduced infiltration of tumor-suppressive lymphocytes in breast cancer through the modulation of intratumoral CXCR3 chemokine release.

## Materials and methods

### Reagents and cell lines

Dulbecco's modified Eagle's medium (DMEM), fetal calf serum (FCS), gentamycin, 4-(2-hydroxyethyl)-1-piperazineethanesulfonic acid (HEPES), and glutamine were from Gibco Life Technologies (Gaithersburg, MD, USA); recombinant human interferon gamma (IFN-γ) and recombinant human TNF-α were from PeproTech (Hamburg, Germany); prostaglandin E_2_, indomethacin, and celecoxib were from Cayman Chemicals (Ann Arbor, MI, USA) and reconstituted in dimethyl sulfoxide (DMSO); acetylsalicylic acid (ASA) was from Sigma (St. Louis, MO, USA) and reconstituted in phosphate buffered saline (PBS); bovine serum albumin was from Sigma (St. Louis, MO, USA). Antibodies to human antigens: monoclonal mouse anti-CXCL9 antibody (clone 49106, R&D Systems, Minneapolis, MN, USA); monoclonal mouse anti-COX-1 (clone COX111, Invitrogen, Camarillo, CA, USA); monoclonal mouse anti-COX-2 (clone CX229, Cayman Chemicals, Ann Arbor, MI, USA); monoclonal mouse anti-glyceraldehyde 3-phosphate dehydrogenase (GAPDH, clone 6C5, Millipore, Billerica, MA, USA); horseradish peroxidase-conjugated goat anti-mouse IgG (Jackson ImmunoResearch, Burlington, ON, USA). All other chemicals were of analytical grade and obtained from Merck (Darmstadt, Germany) or Sigma (St. Louis, MO, USA).

MCF-7 and MDA-MB 231 human breast cancer cell lines (American Type Culture Collection, Manassas, VA, USA) were cultured in a humified 5% CO_2 _atmosphere at 37°C in DMEM supplemented with glutamine, 10% FCS, 10 mM HEPES, and 20 μg/mL gentamycin.

### Human tissue samples and patient characteristics

Fresh-frozen tissues from 60 breast cancer patients who were treated at the Department of Gynecology and Obstetrics, Technical University Munich, between 1991 and 2004 were selected. The study was approved by the local ethics committee, all patients had given written informed consent. All tumors came from nodal-positive, non further metastasized tumors that were estrogen receptor positive (N+, M0, ER+).

### Immunohistochemistry

Immunohistochemistry was performed on 4 μm thick paraffin sections from invasive breast cancer tissue specimens (*n *= 20) obtained from patients treated at the Department of Gynecology and Obstetrics, Technical University Munich. Briefly, sections were deparaffinized by treatment with xylene followed by a graded series of ethanol (100% to 70%) in distilled H_2_O and subjected to heat-induced epitope retrieval in citrate buffer (pH 6.0). Endogenous peroxidase was blocked using 3% H_2_O_2 _in distilled H_2_O, 20 minutes, room temperature, followed by antigen blocking with 5% goat serum in 50 mM Tris-HCl, pH 7.4, 10 minutes, room temperature. The sections were then incubated with mouse anti-CXCL9 diluted to a final concentration of 20 μg/mL in antibody diluent (DAKO, S2022), one hour, room temperature. For detection of primary antibody binding the ZytoChem Plus HRP Broad Spectrum Kit was employed (Zytomed Systems, Berlin, Germany) according to the manufacturer's instruction. Sections were washed thoroughly between incubations and counterstained with hematoxylin.

### Determination of CXCL9 and CXCL10 secretion from breast cancer cells

MCF-7 and MDA-MB 231 cells were plated on 24-well culture plates and grown to 70 to 80% confluency before they were washed in PBS and starved for 24 hours in serum-free medium. The medium was then replaced by serum-free medium and test reagents added as indicated. In case of inhibition studies the cells were preincubated with prostaglandin E_2 _or cyclooxygenase inhibitors (indomethacin, ASA, celecoxib) for 30 minutes before addition of IFN-γ. After 24 hours the supernatants were collected and stored at -20°C until further use. In each experiment, at least six wells were stimulated. Subsequently the culture supernatants were subjected to ELISA for determination of chemokine concentrations. The DuoSet ELISA Kits DY392 and DY266 (R&D Systems, Minneapolis, MN, USA) were used for the determination of CXCL9 and CXCL10, respectively, according to the manufacturer's instructions.

### MTT assay

Viability of cells subjected to cytokines, cyclooxygenase inhibitors, or prostaglandin E_2 _was assessed using the MTT assay (3-(4,5-dimethylthiazol-2-yl)-2,5-diphenyltetrazolium bromide, Sigma-Aldrich, St. Louis, MO, USA). A sample of 5 × 10^3 ^MCF-7 or 7 × 10^3 ^MDA-MB 231 cells were plated into each well of a 96-well plate and cultured for 24 hours. After starving in serum-free medium for another 24 hours, cells were stimulated with the respective stimulants for 24 or 48 hours as indicated in the results section. Then, 20 μL of MTT was added to a final concentration of 200 μg/mL and the cells incubated at 37°C for two hours, followed by the addition of 100 μL DMSO. Absorbance was measured at 590 nm.

### Quantification of CXCL9 concentration in tumor tissue extracts

Breast cancer tumor tissue specimens were obtained at surgery, inspected by a pathologist, and then stored in liquid nitrogen until further use. Tumor tissue homogenates were prepared as described previously [25]. Total protein concentrations were determined applying the BCA Protein Assay Kit (Pierce, Thermo Scientific, Rockford, IL, USA). Tissue Extracts were diluted 1:5 in PBS/1% BSA and then subjected in duplicates to DuoSet ELISA kit (R&D Systems, Minneapolis, MN, USA).

### Western blot analysis

After incubation of the tumor cells with stimulants culture medium was removed and cells washed with ice-cold PBS and immediately lysed in SDS-PAGE sample buffer containing 1% (w/v) Triton X-100, 150 mM NaCl, 1 mM EDTA, 50 mM HEPES, 1 mM sodium vanadate, and complete protease inhibitor cocktail (Roche, Mannheim, Germany). Samples were kept on ice for 20 minutes, subjected to ultrasound (2 × 10 seconds, 4°C), and stored at -20°C until further use. Total protein concentration was determined using the BCA Protein Assay Kit (Pierce, Thermo Scientific, Rockford, IL, USA). Equal amounts of protein (60 μg) were separated by 10% SDS-PAGE and blotted onto polyvinylidene fluoride (PVDF) membranes using a semi-dry transfer apparatus (Biometra, Göttingen, Germany). Blots were blocked with 5% (wt/vol) milk powder (Sigma, St. Louis, MO, USA) containing PBS/0.5% Tween 20 (w/v) (PBS-T) and then incubated at 4°C overnight with primary antibodies (anti-COX-1 0.5 μg/mL, anti-COX-2 0.5 μg/mL, anti-GAPDH 0.1 μg/mL). Following washing with PBS-T and incubation with horseradish peroxidase-coupled anti-mouse IgG (diluted 1:10.000 in 5% milk powder in PBS-T), one hour, room temperature, the blots were washed and the antibody reaction visualized by enhanced chemoluminescent detection (Amersham Biotech, Uppsala, Sweden). Blots were exposed to Kodak X-Omat AR-5 film (Eastman Kodak Co., Rochester, NY, USA).

### Statistical analysis

Results were taken from at least three independent experiments and analyzed using the Mann-Whitney test (SPSS Statistics Software, Zurich, Switzerland, Version 17.0). Results are given as mean ± standard deviation of the mean, if not indicated otherwise. Statistical significance was defined as * *P*≤0.05, ** *P*≤0.005, or *** *P*≤0.0005.

## Results

### Within the tumor microenvironment, CXCL9 is predominantly expressed by breast cancer cells

**Figure 1:**
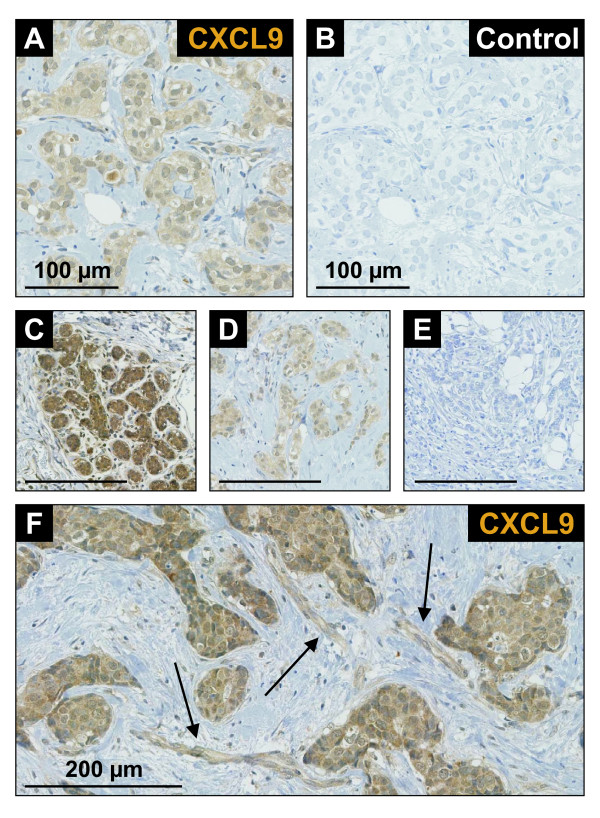
**Expression of the CXCL9 protein in tissue specimens from patients with invasive breast cancer**. **(a) **Representative immunohistochemical staining with the monoclonal antibody to CXCL9 showing staining predominantly in the cytoplasm of tumor cells and in the extracellular matrix. **(b) **No immunoreactivity was seen using isotype matched controls. Different breast cancers showed **(c) **strong, **(d) **weak, or **(e) **no CXCL9 expression. Besides expression in cancer cells and the extracellular matrix, CXCL9 was also found in endothelial cells (arrows in f). Nuclei were counterstained with hematoxylin. *Bars *in c to e, 200 μm.

Datta *et al. *reported that CXCL10 is expressed in breast cancer cells [26]. To find out if this holds true for CXCL9 as well, we performed immunohistochemical analyses of primary breast cancer tissue sections using a monoclonal antibody to CXCL9 (Figure 1a). By this approach we show that CXCL9 is localized predominantly in the cytoplasm of breast cancer cells. The majority of tumor samples (17 of 20) showed CXCL9 immunoreactivity to variable degrees (Figures 1c to 1e). In most samples, CXCL9 protein was also expressed by endothelial cells (15 of 20 samples, Figure 1f) and was found in the extracellular matrix (ECM, 17 of 20 samples), which is supporting data by others showing that CXCR3 ligands may bind to the ECM [27].

### IFN-γ and TNF-α induce CXCR3 ligand release from human breast cancer cell lines

**Figure 2:**
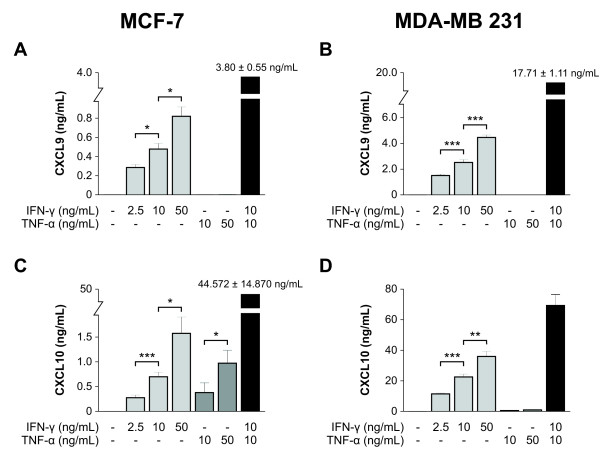
**Induction of CXCR3 binding ligands in human breast cancer cell lines by inflammatory cytokines**. MCF-7 and MDA-MB 231 breast cancer cells were starved in serum-free medium for 24 hours and then exposed for another 24 hours to the IFN-γ and TNF-α concentrations indicated. Supernatants were harvested, and CXCL9 and CXCL10 concentrations determined by ELISA. Results from at least three independent experiments are presented. **(a to d) **In each experiment cells in at least six different wells were stimulated in parallel. IFN-γ induced both chemokines in a dose-dependent manner. **(c and d) **In contrast, TNF-α had a measurable effect on CXCL10 only. **(a to d) **Addition of TNF-α to IFN-γ potentiates the interferon effect. This is more pronounced in MCF-7 than in MDA-MB 231 cells. Statistical significance was defined as * *P*≤0.05, ** *P*≤0.005, or *** *P*≤0.0005.

To study the release of the two chemokines CXCL9 and CXCL10 from breast cancer cells we investigated regulation of protein secretion by supplying the inflammatory cytokines IFN-γ and TNF-α, which are both present in breast cancer tissues and known to induce CXCR3 ligands in other cell types [6,28,29]. CXCL9 and CXCL10 were not released into the culture medium of unstimulated MCF-7 or MDA-MB 231 breast cancer cells, whereas IFN-γ induced both cell types for CXCL9/10 secretion in a dose-dependent manner (Figures 2a to 2d). Stimulation with 50 ng/mL IFN-γ yielded circa five-fold higher CXCL9 and 20-fold higher CXCL10 release by MDA-MB 231 than by MCF-7 cells. TNF-α alone had a measurable effect on CXCL10 release only, but not on CXCL9 (Figures 2c and 2d). The combination of 10 ng/mL TNF-α and 10 ng/mL IFN-γ, however, revealed a strong synergistic effect of both cytokines: CXCL9 secretion increased eight-fold and seven-fold in MCF-7 and MDA-MB 231 cells, respectively, CXCL10 release increased 64-fold and three-fold, respectively, compared with stimulation with 10 ng/mL IFN-γ alone (Figures 2a to 2d).

### PGE_2 _inhibits IFN-γ induced CXCL9 and CXCL10 secretion

**Figure 3:**
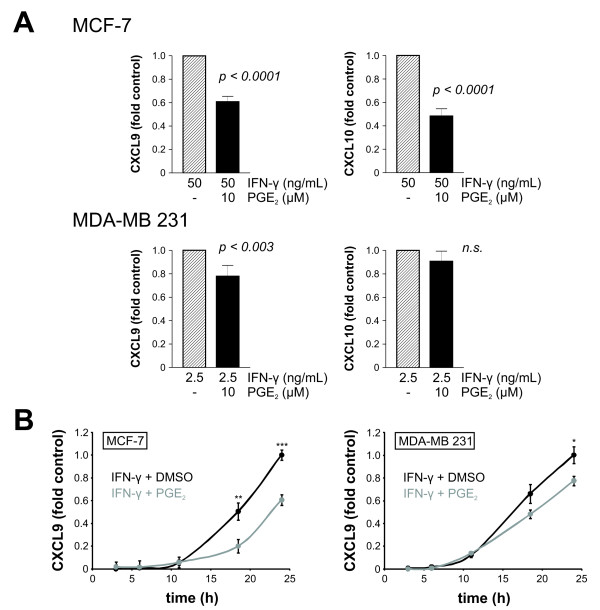
**Suppression of CXCL9 and CXCL10 secretion by PGE_2 _in human breast cancer cell lines**. **(a) **MCF-7 and MDA-MB 231 cells were exposed to 10 μM PGE_2 _for 30 minutes prior to addition of 50 ng/mL or 2.5 ng/mL IFN-γ, respectively (filled bars). Stimulation with IFN-γ and DMSO served as control (dashed bars). After 24 hours, supernatants were collected and CXCR3 ligand concentrations measured by ELISA. Results from at least three independent experiments are presented. In each experiment cells in at least six different wells were stimulated in parallel. In MCF-7 cells, PGE_2 _reduced the CXCL9 secretion to 60.7% ± 4.7% and the CXCL10 secretion to 48.3% ± 6.3%. In MDA-MB 231 cells, this inhibiton was less pronounced and reached statistical significance only for CXCL9 (77.8% ± 9.3%). **(b) **Representative experiment showing the time-dependence of CXCL9 release from MCF-7 (left) and MDA-MB 231 cells (right). The greatest inhibition of CXCL9 release by PGE_2 _(10 μM) was seen at 18 hours after stimulation in MCF-7 and at 24 hours in MDA-MB 231 cells. MCF-7 and MDA-MB 231 cells were stimulated with 50 ng/mL and 2.5 ng/mL IFN-γ, respectively.

To approach our hypothesis that the COX system will have a significant impact on the CXCR3 ligand milieu of breast tumor environment we first investigated the effect of exogenous PGE_2 _on CXCL9 and CXCL10 release. IFN-γ exposed breast cancer cells were used as controls in the subsequent experiments, because IFN-γ is a strong inducer of CXCR3 ligand expression, and the anti-tumor effects of CXCL9 and CXCL10 have been shown to be IFN-γ dependent in mouse models [16,30,31]. Based on the previous results (Figure 2), we stimulated MCF-7 cells with 50 ng/mL IFN-γ and MDA-MB 231 cells with 2.5 ng/mL IFN-γ to induce chemokine concentrations in the sensitivity range of our ELISA kits. Addition of 10 μM PGE_2 _30 minutes prior to IFN-γ stimulation reduced the CXCL9 and CXCL10 secretion by MCF-7 cells approximately 1.6-fold and 2-fold, respectively (Figure 3a). In MDA-MB 231 cells this reduction was less pronounced: using 2.5 ng/mL IFN-γ stimulated cells as control, exposition to 10 μM PGE_2 _30 minutes prior to IFN stimulation reduced the CXCL9 production approximately 1.25-fold (Figure 3a). Time-dependent experiments demonstrated that reduction of CXCL9 by MCF-7 cells was greatest after 18 hours, by MDA-MB 231 cells after 24 hours (Figure 3b).

### Unselective COX inhibitors induce CXCL9 and CXCL10 release from breast cancer cells

**Figure 4:**
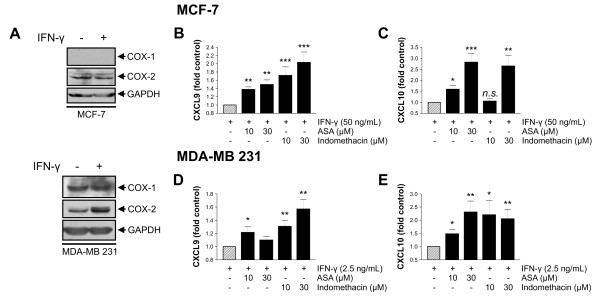
**Induction of CXCR3 ligand release from breast cancer cells by unselective cyclooxygenase inhibitors**. **(a) **Expression of COX-1 and COX-2 isoenzymes in MCF-7 and MDA-MB 231 cells. Representative western blots of lysates prepared from unstimulated and IFN-γ (10 ng/mL, 24 hours) exposed cells using monoclonal antibodies against the two COX isoenzymes. MCF-7 cells showed only weakly inducible COX-2 expression, whereas MDA-MB 231 cells expressed both COX-1 and COX-2, the latter one further induced by IFN-γ. In MCF-7 and in MDA-MB 231 cells, the COX-2 directed antibody shows two bands at 72 and 74 kDa which represent two different glycosylation states [62]. **(b to e) **MCF-7 and MDA-MB 231 cells were preincubated with vehicle or various concentrations of ASA or indomethacin, 30 minutes before IFN-γ (50 ng/mL for MCF-7, 2.5 ng/mL for MDA-MB 231) was added. After 24 hours, cell supernatants were collected and CXCR3 ligand concentrations determined by ELISA. In both cell lines, both unselective COX inhibitors significantly enhance CXCL9 or CXCL10 secretion. **(c and e) **This effect was more pronounced for CXCL10, where the induction was about two- to three-fold of the control value. **(b and d) **For CXCL9, indomethacin was a more potent inducer than ASA. Asterisks mark significant differences compared with the control.

As both MCF-7 and MDA-MB 231 cancer cell lines have been shown to express different COX isoenzymes [32], we expected that blocking endogenous PGE_2 _by COX inhibitors would increase CXCR3 ligand secretion. Whereas both MCF-7 an MDA-MB 231 cells expressed COX-2, COX-1 was expressed by MDA-MB 231 cells only (Figure 4a). In the following, the cells were preincubated with 10 μM or 30 μM of ASA or indomethacin for 30 minutes before IFN-γ was added. Indomethacin increased CXCL9 release in a dose-dependent manner approximately two-fold and 1.6-fold in MCF-7 (Figure 4b) and MDA-MB 231 cells (Figure 4d), respectively, and CXCL10 secretion approximately 2.5-fold in MCF-7 cells (Figure 4c) and 2.0-fold in MDA-MB 231 cells (Figure 4e). ASA increased CXCL9 secretion 1.5-fold and 1.2-fold in MCF-7 and MDA-MB 231 cells, respectively (Figures 4b and 4d) and the CXCL10 secretion 2.8-fold and 2.3-fold (Figures 4c and 4e).

### The COX-2 selective inhibitor celecoxib shows differential effects on CXCR3 ligand secretion

**Figure 5:**
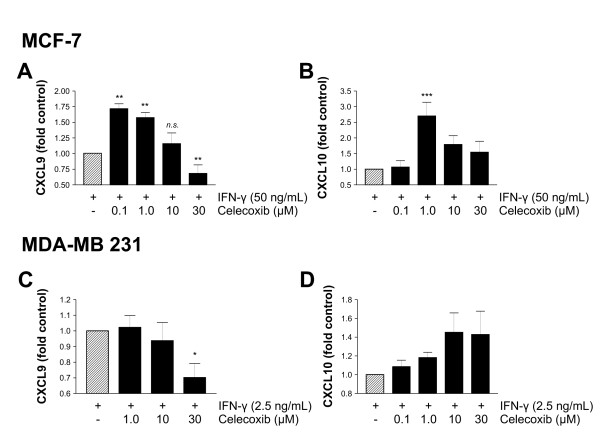
**Differential modulation of CXCR3 ligand release by the COX-2 specific inhibitor celecoxib**. MCF-7 and MDA-MB 231 cells were preincubated with the indicated concentrations of celecoxib 30 minutes prior to IFN-γ addition (50 ng/mL for MCF-7, 2.5 ng/mL for MDA-MB 231). After 24 hours, cell supernatants were collected and CXCR3 ligand concentrations measured by ELISA. **(a and b) **In MCF-7 cells, celecoxib increases CXCL9 and CXCL10 release at low concentrations (0.1 and 1.0 μM), but this increase deminishes at higher concentrations. **(a and c) **At 30 μM celecoxib a significant reduction in CXCL9 secretion was seen. **(d) **The CXCL10 release from MDA-MB 231 cells rises in a dose-dependent manner with increasing celecoxib concentrations. Asterisks mark significant differences compared with the control, if not indicated otherwise.

Subsequently we tested whether the COX-2 specific inhibitor celecoxib would also raise CXCL9 or CXCL10 release from MCF-7 or MDA-MB 231 breast cancer cells. However, the expected increase of CXCL9 in the supernatants was only observed at low concentrations. At elevated concentrations of celecoxib we observed a decline in CXCL9 secretion to approximately 0.7-fold of control in both cell lines by 30 μM celecoxib (Figures 5a and 5c). CXCL10 release was increased in a concentration-dependent manner, but in MCF-7 cells we also observed a decline again when using concentrations above 1.0 μM (Figure 5b). This decline was not observed in MDA-MB 231 cells (Figure 5d). Maximum increase in CXCL9 and CXCL10 secretion from MCF-7 cells was archieved using 0.1 μM celecoxib (approximately 1.7-fold) and 1.0 μM celecoxib (approximately 2.7-fold), respectively.

### Modulation of CXCR3 ligand secretion by COX inhibition or PGE_2 _is not attributable to changes in cell viability

**Figure 6:**
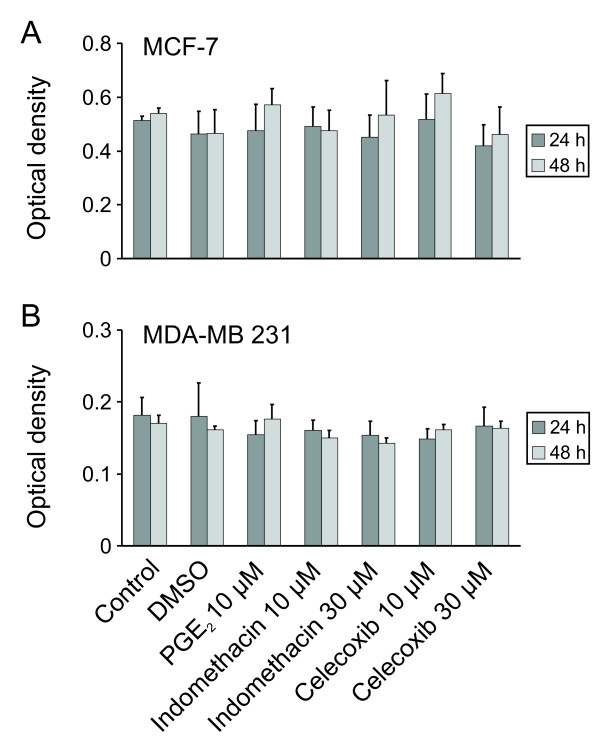
**Influence of reagents on breast cancer cell viability**. MTT assays were performed to determine the influence of the stimulants used in this study on cell viability of MCF-7 and MDA-MB 231 cells. Even at 48 hours no significant difference in cell viablity was observed, showing that the PGE_2 _and COX inhibitor effects are not attributed to altered proliferation or apoptosis.

To rule out the possibility that the changes in CXCL9 or CXCL10 release were due to altered cell proliferation or apoptosis rather than to intracellular regulation, we investigated viability of MCF-7 and MDA-MB 231 cells by MTT assays. Even after 48 hours no significant change in cell viability in the presence of PGE_2 _or unselective or selective COX inhibitors was observed (Figure 6).

### Inverse correlation of COX overexpression and CXCL9 concentration in human breast cancer

**Figure 7:**
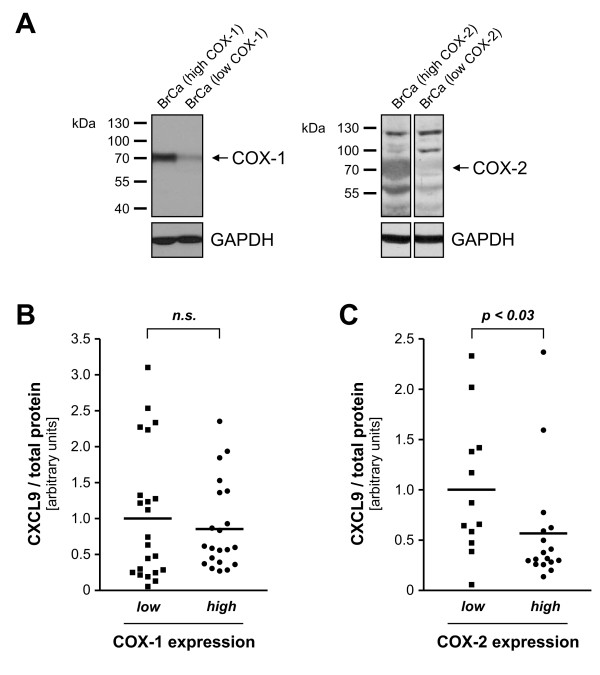
**Correlation between cyclooxygenase and CXCL9 expression in human breast cancer tissue samples**. Homogenates were prepared from deep-frozen breast cancer tissue samples and analyzed for COX-1 and COX-2 expression by western blot analysis. Panel **a **shows representative immunoblots with high (first lane) and low (second lane) expression of both enzymes. For both cyclooxygenase isoenzymes samples were divided into a low and a high expressing group (see text for further details), and CXCL9 concentration was determined in all samples by ELISA. There was a trend towards lower CXCL9 expression in the high COX-1 expressing group (**b**; *n_low _*= 22, *n_high _*= 21), but a significantly reduced CXCL9 concentration in highly COX-2 expressing breast cancers (**c**; *n_low _*= 12, *n_high _*= 17). COX-2 overexpressing breast cancers display only half of the average CXCL9 concentration found in low-expressing cancers (56.5% ± 13.9%). **(b and c) **CXCL9/total protein ratio is presented as arbitrary units with 1.0 set as the arithmetic mean of the low expressing group. Horizontal lines in **b **and **c **represent the arithmetic mean.

To explore whether the observed regulation of CXCL9 by COX could be important also *in vivo*, we prepared tissue extracts from a total of 46 fresh-frozen human breast cancer tumor tissue samples and determined COX-1 and COX-2 expression by western blot and subsequent densiometric analysis (Figure 7a). CXCL9 levels were quantified by ELISA (CXCL9 protein concentrations ranged between less than 0.01 pg and 16.8 pg CXCL9/μg total protein). For each COX isoenzyme samples were divided into a low and a high expressing group: for COX-2 we delineated the samples based on the data by Soslow *et al. *reporting that 65% of breast cancers overexpress COX-2 [33]. As very conflicting data exist for COX-1 overexpression in breast cancers we divided the samples in two equally large groups. With regard to COX-1, there was a trend towards reduced CXCL9 expression in high-expressing tumors (87.8% ± 13.4%, *n_low _*= 22, *n_high _*= 21, Figure 7b). With regard to COX-2, the CXCL9 concentration in the high-expressing group was only half of that of low-expressing tumors (56.5% ± 13.9%, *n_low _*= 12, *n_high _*= 17, *P <*0.026, Figure 7c). Similiar results were obtained when dividing the samples according to the data by Ristimaki *et al. *[34] reporting that 37% of breast cancers overexpress COX-2 (55.0% ± 10.9%, *n_low _*= 17, *n_high _*= 12, *P *= 0.09).

## Discussion

In this study, we have identified the COX pathway as a potential pharmacologic candidate to enhance the intratumoral accumulation of CXCL9 and CXCL10 levels in breast cancer. Earlier studies have highlighted the importance of CXCR3 ligands in recruiting NK cells, as well as CD4^+ ^or CD8^+ ^T lymphocytes to the tumor site [7,30,35,36]. Although the three CXCR3 ligands CXCL9-11 have redundant functions for the most part, for yet unknown reasons CXCL9 emerges as the preferred CXCR3 ligand, mediating lymphocytic infiltration and growth suppression of tumors [30,31]. This is in agreement with clinical studies in human breast cancer identifying CXCL9, rather than CXCL10 or CXCL11, as a potential biomarker for diagnosis of breast cancer and therapy response, suggestive of its protective role in breast cancer biology [14,15,37]. These reasons led us to focus predominantly on CXCL9. Our immunohistochemical breast cancer studies localize CXCL9 to cancer cells (Figure 1). Datta et al. reported similiar findings for CXCL10 [26], emphasizing that these cells are a major source of CXCR3 ligands in the breast tumor microenvironment. However, our results also show CXCL9 expression in endothelial cells. As endothelial cells seem to produce CXCR3 chemokines in response to similiar stimuli as cancer cells they might also participate in modulating immune infiltration in breast cancer [38].

In our study, IFN-γ induced CXCL9 and CXCL10 secretion in a dose-dependent manner, whereas TNF-α induced CXCL10 only. Induction of CXCL10 by TNF-α is in agreement with results obtained in human eosinophils and corneal keratocytes [39,40]. TNF-α potentiated IFN-γ induction of both chemokines, an effect which has been described for other cell types as well and may be ascribed to the synergistic action of transcription factors such as STAT-1α (activated by IFN-γ) and NF-κB (activated by TNF-α) or to the action of transcription coactivators such as CREB binding protein [41]. For the subsequent regulatory experiments on the effects of PGE_2 _and COX inhibitors we decided to use IFN-γ stimulated cells as baseline controls, because it is the most potent inducer of both CXCR3 chemokines and a prerequisite for the immune-mediated tumor-suppressive effects of COX inhibitors in murine breast cancer models [23].

Our results demonstrate that PGE_2 _inhibits, whereas COX inhibitors induce the release of CXCL9 and CXCL10 from breast cancer cells. This is in line with similar results described for epidermoid tumor cells and various kinds of immune cells [42-44]. Both effects were more pronounced in MCF-7 than in MDA-MB 231 cells, although the latter ones expressed higher levels of cyclooxygenases (Figure 4, [32]), so that a higher effect of COX inhibition was expected. Although not the subject of the present study, one may assume that a higher activity of the PGE_2 _prostanoid receptors (EP1-EP4) and their downstream targets in MCF-7 cells is responsible for this difference rather than the higher intrinsic PGE_2 _production in MDA-MB 231 cells. Both cell lines do express all four EP prostanoid receptors [45].

Our findings provide a mechanistic link between the COX pathway and CXCL9/CXCL10 chemokine secretion into the tumor microenvironment of human breast tumors. The inhibition of CXCR3 ligands might be added to the mechanisms by which PGE_2 _promotes tumor escape from the immune system [46]. Several mouse tumor models other than breast cancer have collectively demonstrated that COX inhibition enhances the efficacy of cancer vaccines and impairs tumor growth by raising the number of infiltrating Th1 lymphocytes [17-19,47]. Moreover, these studies have detected an increased expression of Th1 cytokine mRNA, including those of murine CXCL9 and CXCL10 homologs after COX inhibition. Fulton et al. reported that inhibition of COX-1 and COX-2 leads to reduced breast tumor growth and reduced metastatic spread in mice [48]. This inhibition depends on CD4^+ ^and CD8^+ ^T cells in case of local tumor control and NK cell activity and IFN-γ in case of metastatic control [23]. Preincubation of the cancer cells with COX inhibitors prior to injection into the animals is sufficient to cause anti-tumor actitvity, which supports our observation that the tumor cells are the source of the COX-mediated effect [48]. Although determination of chemokine levels was not subject of these studies, our results offer an additional explanation in that enhanced CXCR3 ligand secretion from cancer cells contributes to this immune-mediated anti-cancer effect of COX inhibitors. Clinical data by Denkert et al. further support our interpretation which show a significant correlation between CXCL9 mRNA levels and infiltrating T lymphocytes with favorable chemotherapy response in breast cancer patients [14].

Our findings demonstrate a differential regulation of CXCL9 and CXCL10 secretion by COX inhibitors. Although ASA and indomethacin increased the release of CXCL9 and CXCL10, the COX-2-specific inhibitor celecoxib had this effect only at low concentrations. The mechanisms underlying the inhibitory effect of high concentrations on CXCR3 ligand release remain unclear, but a possible explanation is that the COX inhibitory effects of celecoxib at high concentrations are superimposed by its COX-independent effects, particularly the inactivation of NF-κB signalling [49]. Similiar findings have been reported for the NSAID sulindac, that, in contrast to ASA, impaired CXCL9 mRNA synthesis in mouse macrophages [50]. Moreover, in a murine model of colorectal cancer, celecoxib showed COX-independent anti-tumor activity and even decreased the number of infiltrating lymphocytes [51], consistent with an impairment of CXCR3 ligand release.

Elevated intratumoral COX and PGE_2 _levels in breast cancer are known to be associated with poor outcome and development of distant metastases [34,52,53]. We observed an inverse correlation between COX-2 overexpression and intratumoral CXCL9 concentration and a trend towards lower CXCL9 expression in COX-1 overexpressing breast cancer tissues. This is in line with preclinical data demonstrating that COX-2, rather than COX-1, is inducing PGE_2 _synthesis in breast tumors [54]. In this context, it might be interesting to analyze tumor tissue samples of breast cancer patients that took NSAIDs on a regular basis to see if CXCL9 expression is different from patients who did not, but such investigations were not in focus of the analysis presented.

In conclusion, our results show that COX inhibition is a feasible way to improve immunosurveillance in human breast cancer by inducing intratumoral CXCR3 binding chemokines. To this end, unselective NSAIDs such as indomethacin or aspirin might be more suitable than COX-2-specific agents. The need for COX inhibiton as a component of breast cancer therapy is further endorsed by supporting clinical data. Although the use of NSAIDs in the prevention of breast cancer has been debated for years, albeit with conflicting results [55-58], recent retrospective analyses of large clinical trials have shown that NSAID intake during the course of the cancer disease is associated with a significantly decreased risk of disease recurrence and breast cancer related death [59-61]. Yet, randomized prospective trials are needed to clarify the benefit of COX inhibition in breast cancer therapy and to discover the underlying mechanisms *in vivo*.

## Conclusions

PGE_2 _inhibits IFN-γ-induced release of CXCL9 and CXCL10 in human breast cancer cells. Conversely, the COX inhibitors ASA and indomethacin augment this secretion, whereas the COX-2 specific antagonist celecoxib has differential effects and inhibits CXCR3 ligand release at higher concentrations. In human breast cancer samples, CXCL9 concentrations correlate inversely with COX-2 expression. These results show that unselective COX inhibitors are a feasible pharmacologic way to raise the intratumoral levels of CXCR3 ligands in human breast cancer with the objective to increase tumor infiltration by tumor-suppressive immune cells and improve the outcome of breast cancer therapy.

## Abbreviations

ASA: acetylsalicylic acid; COX: cyclooxygenase; CXCL9: monokine induced by γ-interferon; CXCL10: interferon-γ-inducible protein-10; CXCR3: CXC-chemokine receptor 3; DMEM: Dulbecco's modified eagle medium; DMSO: dimethylsulfoxid; ECM: extracellular matrix; ELISA: enzyme-linked immunosorbend assay; EP: prostanoid receptor for PGE_2_; ER: estrogen receptor; FCS: fetal calf serum; GAPDH: glycerinealdehyde 3-phosphate dehydrogenase; HEPES: 4-(2-hydroxyethyl)-1-piperazineethanesulfonic acid; IFN-γ: interferon gamma; MDSC: myeloid derived suppressor cell; MTT: 3-(4,5-dimethylthiazol-2-yl)-2,5-diphenyltetrazolium bromide; NF-κB: nuclear factor 'kappa-light-chain-enhancer' of activated B-cells; NSAID: non steroidal anti-inflammatory drug; PBS: phosphate buffered saline; PGE_2_: prostaglandin E_2_; PVDF: polyvinylidenfluorid; SDS-PAGE: sodium dodecyl sulfate polyacrylamide gel electrophoresis; STAT-1α: signal transducer and activator of transsription 1α; TNF-α: tumor necrosis factor alpha.

## Competing interests

The authors declare that they have no competing interests.

## Authors' contributions

HB coordinated the studies, contributed to all of the experiments, and drafted the manuscript. SK and AS performed the stimulation experiments, western blot, and MTT assays. USB provided the patient collectives. CC and SA contributed to the immunohistochemical localization experiments. MK and MS participated in the design of the study, and supervised the research project. All authors read and approved the final manuscript.
